# Infected Wound Secondary to a Hyena Bite in a Patient From Rural South Sudan: A Case Report

**DOI:** 10.7759/cureus.105576

**Published:** 2026-03-21

**Authors:** Langa James Oriho, Dario Kuron Lado, Micheal Yoana Tangun

**Affiliations:** 1 Department of Surgery, Jimma University, Jimma, ETH; 2 Department of Surgery, University of Juba, Juba, SSD; 3 Department of Surgery, Juba Teaching Hospital, Juba, SSD

**Keywords:** global surgery, hyena attack, hyena bite, infected wound, sepsis

## Abstract

Animal bites, especially hyena bites, can lead to severe injuries and cause significant morbidity and disabilities to a patient, most commonly in low- and middle-income countries. In South Sudan, the most common type of hyena is the Spotted Hyena (*Crocuta crocuta*).

The management of severe injuries due to a hyena bite is multi-disciplinary and challenging in low- and middle-income countries (LMIC). Acute emergency conditions like active bleeding and sepsis should be treated, the wound should be cleaned, debrided from devitalized tissue, and reconstructive procedures should be done when the wound is clean. Hyena bite injuries can range from mild to severe injuries.

We present a case of an 11-year-old female patient who presented to our emergency room with a wound on the left hand and right buttock after she was attacked by a hyena three days prior and was referred from Tonj Hospital, Warrap State. The patient underwent debridement as well as second-look debridement and is awaiting reconstructive surgery. This case report highlighted the emergency management of complex hyena injuries and the importance of prompt intervention in the overall outcomes.

## Introduction

Major injuries caused by animal bites are a surgical emergency [1]. A surveillance study in Uganda has shown that the most common cases are dog bites, followed by snakes, donkeys, and, in some cases, wild animals (hyenas, crocodiles, monkeys, etc.) [1-3]. Hyena injuries are rare, and only a handful of case reports and case series describe incidences [3-8]. In Sub-Saharan Africa, the most common type of hyena is the spotted hyena (*Crocuta crocuta*) [3]. Other types of hyenas are the Striped, Brown Hyena, and Aardwolf [4]. The spotted hyena hunts and scavenges in its natural habitat. In northern Ethiopia, spotted hyenas are more commonly found in human-dominated settings than in their natural habitats [3,4]. The hyenas mostly target vulnerable victims, usually children, and sleeping humans during the evening or night. Injuries due to hyena attacks are severe and mostly require complex surgical intervention [5]. Wound infection is one of the complications of a hyena bite, which is due to bacterial flora in the oral cavity and teeth of the hyena [9,10]. Management of hyena bites mostly involves wound cleaning/debridement with or without reconstructive procedures [3,10]. Here is a case of an 11-year-old female patient who presented with an infected wound due to a hyena bite.

## Case presentation

**Figure 1:**
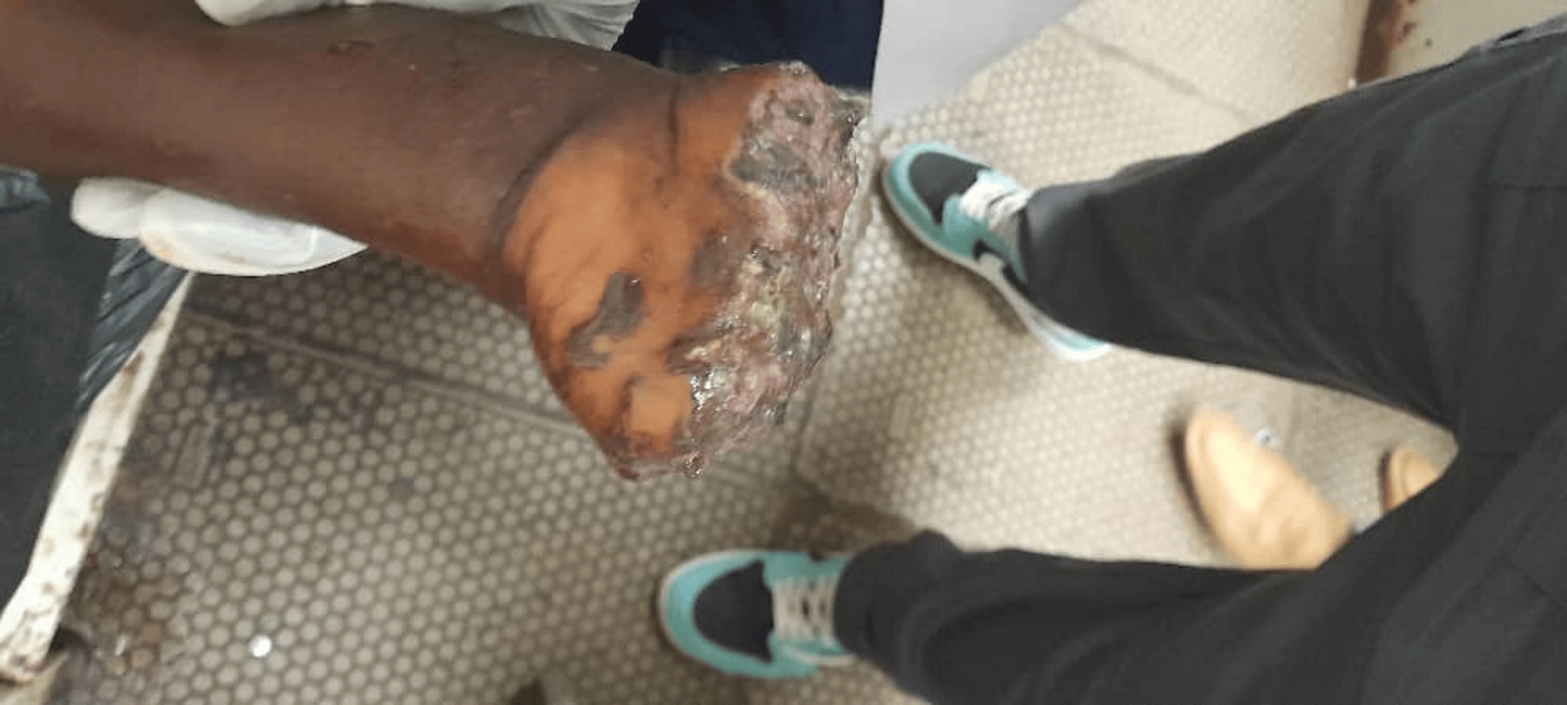
Traumatic, amputated left hand of the patient

**Figure 2:**
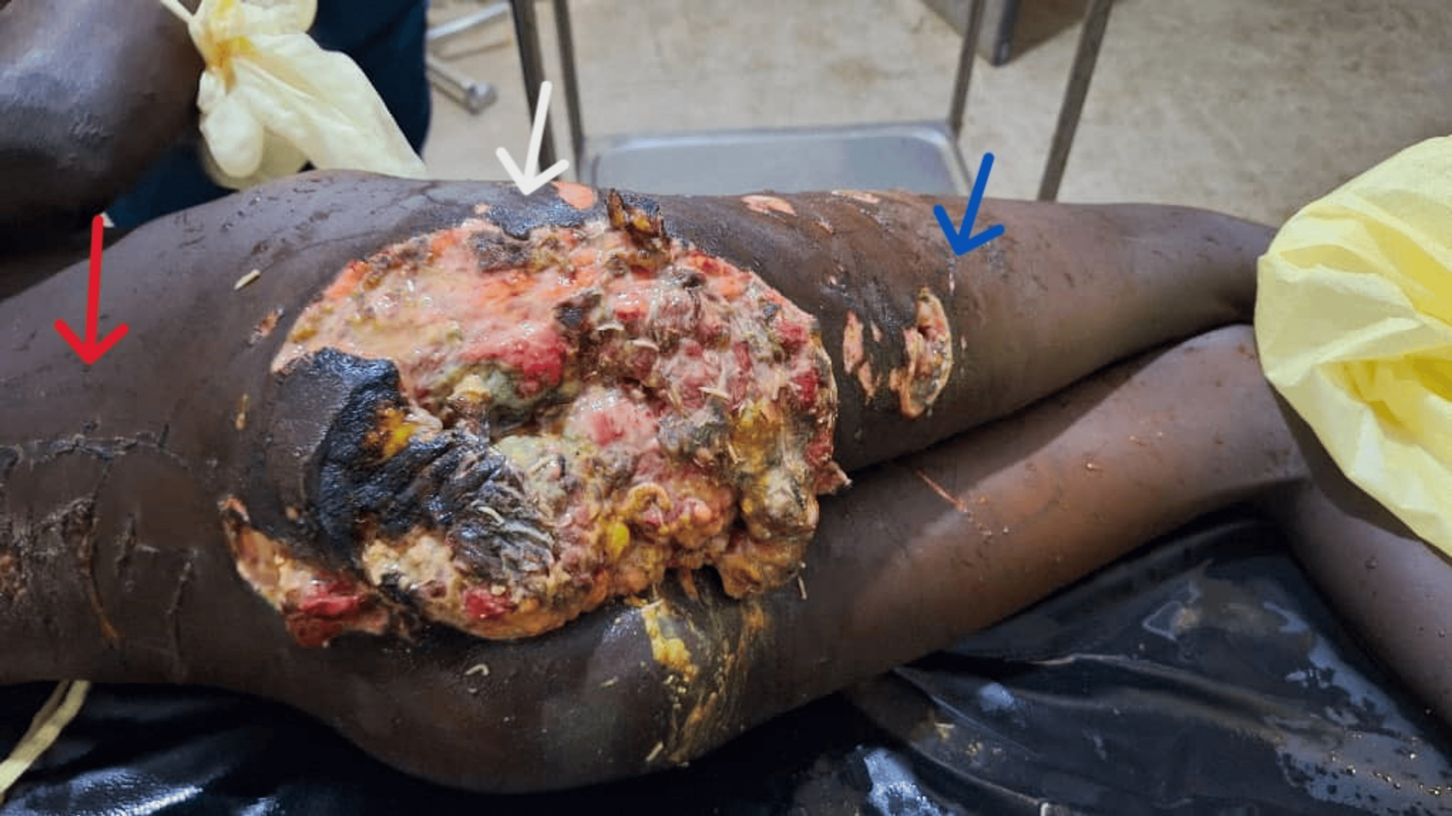
Infected wound in the right buttock (white arrow), the back (red arrow), and the right posterior thigh (blue arrow)

An 11-year-old female patient presented to the emergency room of Juba Teaching Hospital with multiple wounds (left hand, back side, right buttock, and right thigh) after she was attacked by a hyena 3 days prior. She was referred from Tonj County Hospital, Warrap State, South Sudan. According to the patient, she was attacked at night by a hyena near the compound of her house. On presentation, she complained of pain and discharge from wound sites. Upon examination, the patient was conscious (Glasgow coma scale 15/15) and in pain. Her vital signs were blood pressure of 100/80 millimeters mercury (mmHg), pulse rate of 137 beats per minute (bpm), respiratory rate of 28 breaths per minute, a temperature of 37.1 degrees Celsius, and oxygen saturation was 96% with atmospheric air. Her conjunctivae were pale. In the musculoskeletal system, there was a traumatic amputation of the left hand, involving all the fingers at the level of the metacarpophalangeal (MCP) joints, as seen in Figure 1. Also on examination, there was a 20 cm * 18 cm lacerated wound of the right gluteal area; the wound was darkly discolored with pus discharge and offensive smell, 5 cm from the anal orifice. There is a 6 cm * 4 cm laceration of the posterior right thigh, and there were scratches on the back, as seen in Figure 2. A digital rectal examination showed no lesion, no blood, and no connection between the wound and rectum. With a diagnosis of an infected wound secondary to a hyena bite, she was resuscitated with 2 liters of normal saline within one hour, and she produced adequate urine output.

Her complete blood count (CBC) was as follows: white blood cell (WBC) count 25,600/ microliter (μl) (3,000-15,000 μl), neutrophil 80.0% (37-72%), hemoglobin 10.3 gram per deciliter (g/dl) (8-17 g/dl), hematocrit - 34.9% (25-50%), and platelets 150,000/μl (150,000-450,000 μl).

**Figure 3:**
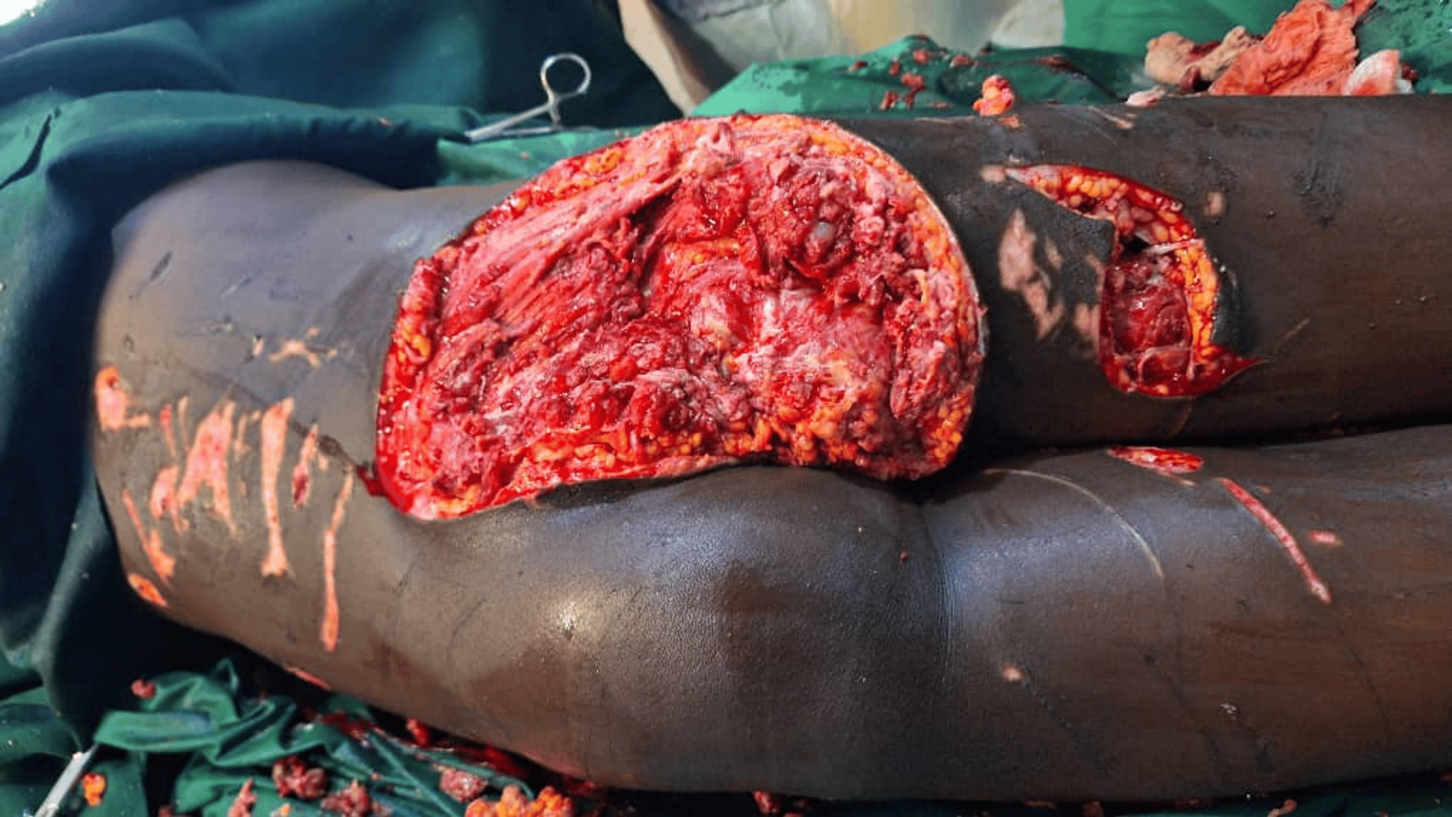
Intraoperative picture following the debridement

Antibiotics were administered, including ceftriaxone and metronidazole. Analgesia was provided (paracetamol infusion and diclofenac injection). After consulting the orthopedic department, she was taken to the operating room for urgent debridement after she was transfused one unit of whole blood. The patient underwent debridement. Intraoperatively, the infected wound involved the gluteus maximus, medius, and minimus muscles; part of these muscles were necrotic, and there were pus pockets between the muscle fascia. However, the sciatic nerve with its branches was not involved. The devitalized tissues were debrided, and the wound was washed with hydrogen peroxide and normal saline (Figure 3), dressed with antibiotic ointment. Jointly, during the procedure, the orthopedic team performed a corrective amputation of the left hand.

**Figure 4:**
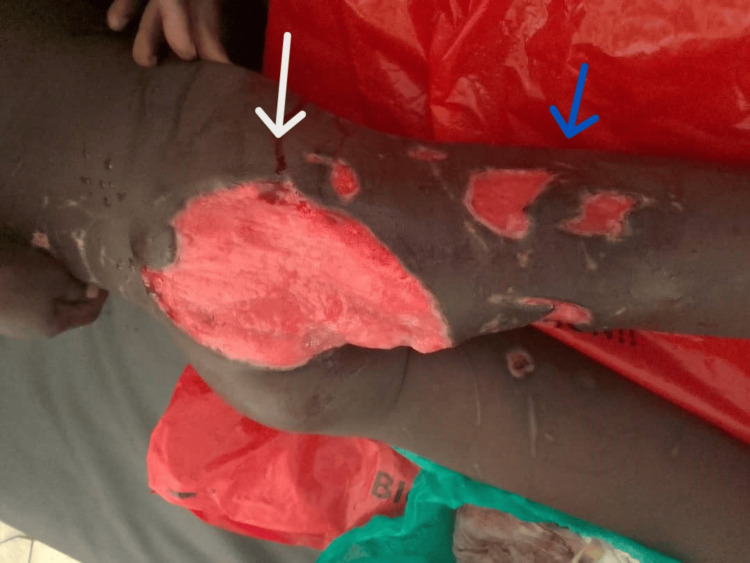
The right gluteal wounds (white arrow) and the right posterior thigh wounds (blue arrow) two weeks after second-look debridement

Postoperatively, the patient was transfused with two units of whole blood and has been stable. After 48 hours, the patient underwent second-look debridement. The routine wound dressing consisted of antibiotic ointment (tetracycline and nitrofurazone) after cleaning with normal saline; no negative-pressure wound therapy was used. Subsequently, after two weeks of wound management, the wounds were clean and granulating (Figure 4), awaiting reconstructive surgery to be performed by a plastic surgeon. The challenge encountered was the unavailability of a plastic surgeon at the teaching hospital; the patient was sent to a plastic surgeon at another center. After discharge, the patient was lost to follow-up.

## Discussion

Animal bites are a global public health concern [1]. Major injuries from animal bites usually need urgent medical and surgical intervention [3,5]. According to the literature, dog bites are the most common animal bites worldwide; other domestic or wild animals can also cause bite injuries [10]. Animal bites due to hyenas are rare and occur in areas where hyena species reside [3-8]. In rural Africa, hyenas came into proximity to humans due to population expansion into their natural habitat [9]. Hyena attacks cause major injuries due to the hyena's grabbing and ripping the victim's body with substantial force. It usually attacks the head and neck (spinal cord area) to cause submission. This pattern of attack, which involves inflicting injuries to the face and neck, is demonstrated in large cats and dogs [7]. In our patient, the pattern of injuries sustained is consistent with running away from the hyena. There is always a risk of infection from a hyena bite and a significant risk of rabies [3-5]. A study showed that the oral cavity of adult spotted hyena is mostly populated by *Pasteurellaceae*, *Leptotrichiaceae*, and *Porphyromonadaceae* species [9]. Hyenas rarely show symptoms despite high exposure to rabies. Patients with a hyena bite should receive rabies and tetanus vaccines in accordance with available guidelines [1,4]. Our patient did not receive the rabies vaccine at the government hospital because of unavailability. She was advised to get the vaccine from private centers.

Injuries due to hyenas are usually major and complex due to the severity and fatality of the attacks [5,7]. Major injuries due to hyenas can be complex facial injuries, limbs, trunk, or injuries to the genital area [3-8]. Management of the major injuries due to a hyena bite starts with approaching the trauma patient using ATLS (Advanced Trauma Life Support); the current ATLS 11 edition algorithm now follows xABCDE, where x emphasizes emergent external hemorrhage control [11]. We should identify injuries, address the life-threatening conditions, and send for investigations that will help us, such as a complete blood count and blood grouping. In some cases, an X-ray is important to rule out fractures or joint involvement, and a CT scan or CT angiography is essential for head and neck injuries or vascular injuries [1,3]. After initial management, we focus on the soft tissue and the prevention of local and/or systemic infection [7]. The wound must be cleaned and debrided [3-5,10], and sepsis should be managed according to the Surviving Sepsis Campaign guidelines [12]. Empirical antibiotics should be used after samples from blood and other bodily fluids are sent for cultures and sensitivity. Prophylactic antibiotics for hyena bites are essential, but controversial [7]. A Cochrane review showed a decrease in infection after human bites, but not after cat and dog bites, when prophylactic antibiotics were used [13]. However, our patient received empirical antibiotics; no samples were sent for cultures and sensitivity because they were not available at the hospital at that time. The sepsis was managed, and we controlled the wound infection; moreover, the patient needed further reconstructive procedures by a plastic surgeon, and we had to refer the patient to another center. Complex injuries caused by hyena injuries require a multidisciplinary team, and securing a team of specialists is challenging in low-income countries [3]. In our case, we involved a team of surgeons, physiotherapists, and psychologists. A plastic surgeon was not available to perform a complex reconstructive procedure. Management of complex injuries caused by hyena is usually challenging in resource-limited settings [3], and policymakers and stakeholders should invest more in health care so that such cases can be managed in teaching hospitals. In conclusion, prompt surgical intervention prevented life-threatening complications; any delay would have led to a catastrophic outcome. Patients in low-income countries deserve accessible/quality surgical services, as do those in high-income countries; it is a health equity issue.

## Conclusions

A hyena bite can cause severe injuries that need urgent treatment. Patients with these major injuries should undergo a systematic approach to the management of trauma patients. Prompt surgical intervention is vital to prevent life-threatening complications. The management of hyena bites is multidisciplinary and challenging in low- and middle-income countries (LMIC).
